# Octa-μ_3_-selenido-penta­kis­(tri­ethyl­phos­phane-κ*P*)(tri­methyl­aceto­nitrile-κ*N*)-*octa­hedro*-hexa­rhenium(III) bis­(hexa­fluorido­anti­monate) tri­methyl­aceto­nitrile monosolvate

**DOI:** 10.1107/S1600536814011982

**Published:** 2014-06-04

**Authors:** YiXin Ren, Andrea M. Bruck, Lisa F. Szczepura

**Affiliations:** a Department of Chemistry, Campus Box 4160, Illinois State University, Normal, IL 61790-4160, USA

## Abstract

The crystal structure of the title compound, [Re_6_Se_8_{NCC(CH_3_)_3_}(Et_3_P)_5_](SbF_6_)_2_·NCC(CH_3_)_3_, contains a face-capped octa­hedral [Re_6_(μ_3_-Se)_8_]^2+^ cluster core. The pseudo-centrosymmetric [Re_6_Se_8_]^2+^ cluster core is bonded through the Re atoms to five tri­ethyl­phosphane ligands and one tri­methyl­aceto­nitrile ligand. No significant interactions are observed between the cationic cluster, the SbF_6_^−^ anions and the trimethylacetonitrile solvent molecule.

## Related literature   

For the preparation of site-differentiated rhenium chalcogenide cluster complexes, see: Zheng *et al.* (1997 ▶); Willer *et al.* (1998 ▶); Szczepura *et al.* (2010 ▶). For the structure of the first [Re_6_Se_8_]^2+^-based cluster complex containing a nitrile ligand (MeCN), see: Zheng *et al.* (1997 ▶). For the crystal structures of other rhenium chalcogenide cluster complexes, see: Long *et al.* (1996 ▶); Brylev *et al.* (2003 ▶); Dorson *et al.* (2009 ▶) and for additional [Re_6_Se_8_]^2+^-based complexes containing nitrile ligands, see: Zheng & Holm (1997 ▶); Zheng *et al.* (1999 ▶); Durham *et al.* (2012 ▶); Wilson *et al.* (2014 ▶). For the reactivity of transition metal nitrile complexes, see: Endres (1987 ▶).
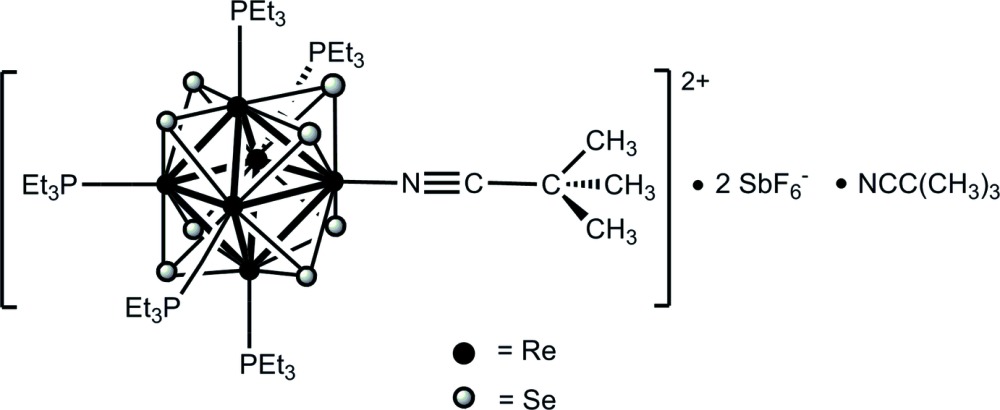


## Experimental   

### 

#### Crystal data  

[Re_6_Se_8_(C_5_H_9_N)(C_6_H_15_P)_5_](SbF_6_)_2_·C_5_H_9_N
*M* *_r_* = 2977.39
Triclinic, 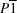

*a* = 14.3341 (10) Å
*b* = 16.6498 (11) Å
*c* = 17.0533 (11) Å
α = 82.157 (1)°
β = 72.859 (1)°
γ = 71.608 (1)°
*V* = 3686.3 (4) Å^3^
*Z* = 2
Mo *K*α radiation
μ = 14.65 mm^−1^
*T* = 173 K
0.56 × 0.30 × 0.25 mm

#### Data collection  

Bruker APEXII CCD diffractometer
Absorption correction: integration (*SADABS*; Bruker, 2008 ▶) *T*_min_ = 0.020, *T*_max_ = 0.104
33157 measured reflections
16902 independent reflections
14674 reflections with *I* > 2σ(*I*)
*R*_int_ = 0.018

#### Refinement  

*R*[*F* ^2^ > 2σ(*F* ^2^)] = 0.024
*wR*(*F* ^2^) = 0.056
*S* = 1.08
16902 reflections
676 parameters
H-atom parameters constrained
Δρ_max_ = 3.10 e Å^−3^
Δρ_min_ = −1.92 e Å^−3^

### 

Data collection: *APEX2* (Bruker, 2008 ▶); cell refinement: *SAINT* (Bruker, 2008 ▶); data reduction: *SAINT*; program(s) used to solve structure: *DIRDIF08* (Beurskens *et al.*, 2008 ▶); program(s) used to refine structure: *SHELXL97* (Sheldrick, 2008 ▶); molecular graphics: *ORTEP-3 for Windows* (Farrugia, 2012 ▶); software used to prepare material for publication: *WinGX* (Farrugia, 2012 ▶).

## Supplementary Material

Crystal structure: contains datablock(s) I, ISU1305. DOI: 10.1107/S1600536814011982/gk2611sup1.cif

Structure factors: contains datablock(s) I. DOI: 10.1107/S1600536814011982/gk2611Isup2.hkl

CCDC reference: 1004865

Additional supporting information:  crystallographic information; 3D view; checkCIF report

## Figures and Tables

**Fig. 1.**
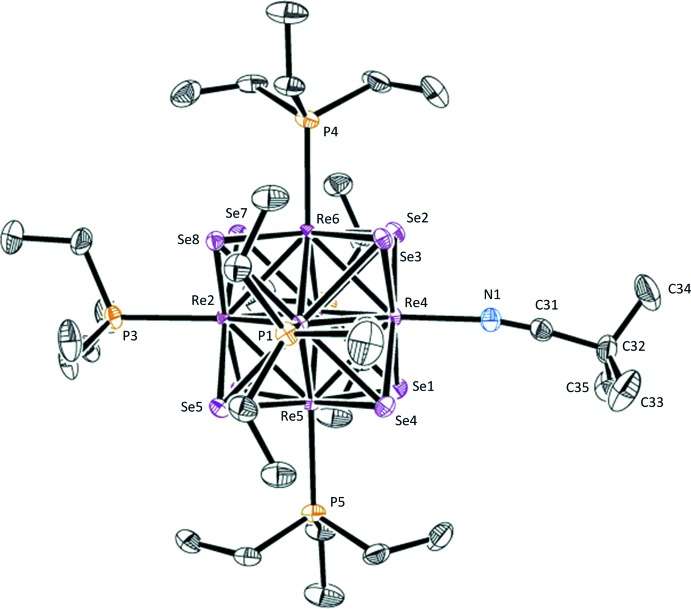
Fig. 1. ORTEP diagram of the [Re6Se8(PEt3)5(NCC(CH3)3)]2+ cation with hydrogen atoms omitted for clarity. Non-hydrogen atoms are represented by ellipsoids at the 50% probability level.

## supplementary crystallographic information

### S1. Comment

Discrete clusters based on the [Re_6_Q_8_]^2+^ (Q = S or Se) are relatively robust and undergo substitution chemistry (Zheng *et al.*, 1997; Willer *et al.*, 1998; Szczepura *et al.*, 2010). Substitution of the terminal halide ligands with inert phosphane ligands on these cores has greatly facilitated research in this area. Because nitrile ligands are often readily substituted by stronger ligands, complexes containing organonitrile ligands are typically used as precursors in the preparation of coordination complexes (Endres, 1987). The present paper describes the crystal structure of the title compound from single-crystal X-ray diffraction data. The cluster complex shows core bond lengths (Re–Re) and (Re–Se) and angles (Re–Re–Re, Re–Re–Se, Se–Re–Se, and Re–Se–Re) are within the range typically found for [Re_6_Se_8_]^2+^ based cluster complexes (Long *et al.*, 1996; Brylev *et al.*, 2003; Dorson *et al.*, 2009). The observed Re–P (2.414–2.512 Å) and Re—N (2.125 Å) bond lengths both fall within ranges previously observed for [Re_6_Se_8_]^2+^ based cluster complexes containing terminal triethylphosphane as well as nitrile ligands (Zheng *et al.*, 1997; Zheng *et al.*, 1999; Zheng & Holm, 1997; Durham *et al.*, 2012; Wilson *et al.*, 2014). As with other nitrile ligands bound to rhenium selenide cores, the Re—N—C bond angle of 170.0 (4)° indicates an arrangement that is close to linear.

### S2. Experimental

The [Re_6_Se_8_(PEt_3_)_5_I]I complex was obtained according to a previously published procedure (Zheng *et al.*, 1997). The title compound was prepared by dissolving [Re_6_Se_8_(PEt_3_)_5_I]I (300 mg, 0.115 mmol) in 15 ml of CH_2_Cl_2_. Separately, 94.2 mg of AgSbF_6_ (0.274 mmol) was dissolved in 540 µ*L* of trimethylacetonitrile. These solutions were combined, covered with aluminium foil, and stirred at room temperature for 3 h under N_2_ gas. The resulting mixture was then filtered through Celite; the filtrate was reduced to dryness on the Schlenk line. The remaining residue was dissolved in 1.5 ml of CH_2_Cl_2_ and added drop-wise into Et_2_O to afford a solid. Single crystals of [Re_6_Se_8_(PEt_3_)_5_(NCC(CH_3_)_3_)](SbF_6_)_2_·NCC(CH_3_)_3_ suitable for X-ray analysis were grown *via* the vapor diffusion technique using trimethylacetonitrile and Et_2_O at -20 °C. The single crystals obtained are of orange color. MS (ESI(+)): m/*z* 1212.0 ([Re_6_Se_8_(PEt_3_)_5_(NCC(CH_3_)_3_)]^2+^). Anal. Calcd for C_35_H_84_F_12_NP_5_Re_6_Sb_2_Se_8_: C, 14.52; H, 2.93; N, 0.48. Found: C, 14.44; H, 2.82; N, 0.49.

### S3. Refinement

The highest peak of 3.10 e-/Å^3^ is 0.88 Å from atom Sb2 and the deepest hole of -1.31 e-/Å^3^ is 0.32 Å from Sb2. All H-atoms were refined using constrained model.

### Crystal data

**Table:**

[Re_6_Se_8_(C_5_H_9_N)(C_6_H_15_P)_5_](SbF_6_)_2_·C_5_H_9_N	*Z* = 2
*M**_r_* = 2977.39	*F*(000) = 2708
Triclinic, *P*1	*D*_x_ = 2.682 Mg m^−^^3^
Hall symbol: -P 1	Mo *K*α radiation, λ = 0.71073 Å
*a* = 14.3341 (10) Å	Cell parameters from 9836 reflections
*b* = 16.6498 (11) Å	θ = 2.2–27.5°
*c* = 17.0533 (11) Å	µ = 14.65 mm^−^^1^
α = 82.157 (1)°	*T* = 173 K
β = 72.859 (1)°	Prism, orange
γ = 71.608 (1)°	0.56 × 0.30 × 0.25 mm
*V* = 3686.3 (4) Å^3^	

### Data collection

**Table:**

Bruker APEXII CCD diffractometer	14674 reflections with *I* > 2σ(*I*)
Graphite monochromator	*R*_int_ = 0.018
ω scans	θ_max_ = 27.5°, θ_min_ = 1.3°
Absorption correction: integration (*SADABS*; Bruker, 2008)	*h* = −18→18
*T*_min_ = 0.020, *T*_max_ = 0.104	*k* = −21→21
33157 measured reflections	*l* = −22→22
16902 independent reflections	

### Refinement

**Table:**

Refinement on *F*^2^	Primary atom site location: structure-invariant direct methods
Least-squares matrix: full	Secondary atom site location: difference Fourier map
*R*[*F*^2^ > 2σ(*F*^2^)] = 0.024	Hydrogen site location: inferred from neighbouring sites
*wR*(*F*^2^) = 0.056	H-atom parameters constrained
*S* = 1.08	*w* = 1/[σ^2^(*F*_o_^2^) + (0.015*P*)^2^ + 11.3814*P*] where *P* = (*F*_o_^2^ + 2*F*_c_^2^)/3
16902 reflections	(Δ/σ)_max_ = 0.023
676 parameters	Δρ_max_ = 3.10 e Å^−^^3^
0 restraints	Δρ_min_ = −1.92 e Å^−^^3^

### Special details

**Table:**

Geometry. All s.u.'s (except the s.u. in the dihedral angle between two l.s. planes) are estimated using the full covariance matrix. The cell s.u.'s are taken into account individually in the estimation of s.u.'s in distances, angles and torsion angles; correlations between s.u.'s in cell parameters are only used when they are defined by crystal symmetry. An approximate (isotropic) treatment of cell s.u.'s is used for estimating s.u.'s involving l.s. planes.

### Fractional atomic coordinates and isotropic or equivalent isotropic displacement parameters (Å^2^)

**Table:**

	*x*	*y*	*z*	*U*_iso_*/*U*_eq_	
C1	1.1228 (4)	0.5555 (3)	0.5509 (3)	0.0418 (13)	
H1A	1.1501	0.5042	0.5195	0.063*	
H1B	1.0646	0.5502	0.5969	0.063*	
H1C	1.1759	0.5629	0.5721	0.063*	
C2	1.0880 (4)	0.6322 (3)	0.4951 (3)	0.0320 (11)	
H2A	1.0359	0.6229	0.4729	0.038*	
H2B	1.1471	0.6354	0.448	0.038*	
C3	1.1370 (3)	0.7479 (4)	0.5787 (3)	0.0320 (11)	
H3A	1.114	0.8051	0.6003	0.038*	
H3B	1.1473	0.7064	0.6251	0.038*	
C4	1.2399 (4)	0.7375 (5)	0.5148 (4)	0.0511 (16)	
H4A	1.2889	0.746	0.5401	0.077*	
H4B	1.2317	0.7795	0.4692	0.077*	
H4C	1.2651	0.6804	0.494	0.077*	
C5	1.0268 (4)	0.8076 (3)	0.4538 (3)	0.0282 (10)	
H5A	1.0926	0.7909	0.4112	0.034*	
H5B	0.9734	0.8014	0.4312	0.034*	
C6	1.0030 (4)	0.9008 (3)	0.4699 (3)	0.0379 (12)	
H6A	1.0005	0.935	0.4186	0.057*	
H6B	1.0563	0.9083	0.4908	0.057*	
H6C	0.9369	0.9189	0.5106	0.057*	
C7	0.7390 (4)	1.0918 (3)	0.6885 (3)	0.0298 (10)	
H7A	0.8076	1.0669	0.652	0.036*	
H7B	0.7127	1.1504	0.6669	0.036*	
C8	0.7500 (5)	1.0961 (3)	0.7741 (3)	0.0447 (14)	
H8A	0.7961	1.1298	0.7708	0.067*	
H8B	0.7781	1.0386	0.7957	0.067*	
H8C	0.683	1.1224	0.8107	0.067*	
C9	0.5643 (5)	1.0300 (3)	0.5562 (4)	0.0454 (14)	
H9A	0.5635	1.0482	0.4992	0.068*	
H9B	0.4968	1.0547	0.5929	0.068*	
H9C	0.5818	0.9681	0.5623	0.068*	
C10	0.5307 (4)	1.0819 (3)	0.7486 (4)	0.0373 (12)	
H10A	0.4826	1.0514	0.7462	0.045*	
H10B	0.5359	1.0754	0.8059	0.045*	
C11	0.4850 (5)	1.1758 (3)	0.7288 (4)	0.0510 (15)	
H11A	0.4182	1.1969	0.768	0.077*	
H11B	0.477	1.1834	0.6729	0.077*	
H11C	0.5304	1.2075	0.7329	0.077*	
C12	0.6435 (4)	1.0595 (3)	0.5783 (3)	0.0355 (12)	
H12A	0.626	1.122	0.5701	0.043*	
H12B	0.7108	1.0356	0.5395	0.043*	
C13	0.5609 (5)	0.5876 (4)	0.5062 (4)	0.0490 (15)	
H13A	0.5699	0.5264	0.5096	0.074*	
H13B	0.5938	0.6048	0.4502	0.074*	
H13C	0.4879	0.6181	0.5199	0.074*	
C14	0.4643 (3)	0.7743 (3)	0.5917 (3)	0.0266 (10)	
H14A	0.4541	0.8354	0.5956	0.032*	
H14B	0.4403	0.7684	0.5445	0.032*	
C15	0.3978 (4)	0.7430 (4)	0.6699 (3)	0.0391 (12)	
H15A	0.3263	0.776	0.6763	0.059*	
H15B	0.4193	0.75	0.7174	0.059*	
H15C	0.4053	0.683	0.6662	0.059*	
C16	0.6459 (4)	0.7546 (3)	0.4635 (3)	0.0291 (10)	
H16A	0.5996	0.7489	0.4327	0.035*	
H16B	0.6403	0.8156	0.4619	0.035*	
C17	0.7553 (4)	0.7075 (4)	0.4187 (3)	0.0381 (12)	
H17A	0.7719	0.731	0.3621	0.057*	
H17B	0.7617	0.6472	0.4181	0.057*	
H17C	0.8025	0.7142	0.4472	0.057*	
C18	0.6094 (4)	0.6083 (3)	0.5667 (3)	0.0320 (11)	
H18A	0.6824	0.5754	0.553	0.038*	
H18B	0.577	0.5884	0.6225	0.038*	
C19	0.4316 (4)	0.8789 (3)	0.9937 (3)	0.0350 (11)	
H19A	0.3753	0.8733	1.0424	0.042*	
H19B	0.4877	0.8838	1.0137	0.042*	
C20	0.3940 (4)	0.9598 (3)	0.9445 (4)	0.0440 (14)	
H20A	0.3707	1.0084	0.9792	0.066*	
H20B	0.3372	0.9564	0.9255	0.066*	
H20C	0.4497	0.9669	0.897	0.066*	
C21	0.3689 (3)	0.7825 (3)	0.9000 (3)	0.0313 (11)	
H21A	0.3583	0.8296	0.8583	0.038*	
H21B	0.3868	0.729	0.8723	0.038*	
C22	0.2677 (4)	0.7912 (5)	0.9667 (4)	0.0512 (16)	
H22A	0.2146	0.791	0.9415	0.077*	
H22B	0.248	0.8446	0.9938	0.077*	
H22C	0.2761	0.7437	1.0073	0.077*	
C23	0.4835 (4)	0.7007 (3)	1.0184 (3)	0.0385 (12)	
H23A	0.5385	0.701	1.0423	0.046*	
H23B	0.4184	0.7163	1.0619	0.046*	
C24	0.5023 (5)	0.6109 (4)	0.9952 (4)	0.0537 (16)	
H24A	0.504	0.5725	1.0441	0.081*	
H24B	0.5677	0.5935	0.9536	0.081*	
H24C	0.4473	0.6089	0.9731	0.081*	
C25	0.8896 (4)	0.9178 (3)	0.9126 (3)	0.0351 (11)	
H25A	0.9174	0.9402	0.8567	0.042*	
H25B	0.8155	0.947	0.9292	0.042*	
C26	0.9392 (5)	0.9411 (4)	0.9713 (4)	0.0581 (18)	
H26A	0.9253	1.0027	0.9693	0.087*	
H26B	1.0131	0.9142	0.9546	0.087*	
H26C	0.9109	0.9211	1.0274	0.087*	
C27	0.8660 (4)	0.7665 (3)	1.0143 (3)	0.0315 (11)	
H27A	0.8763	0.7048	1.0147	0.038*	
H27B	0.9113	0.7746	1.0447	0.038*	
C28	0.7571 (4)	0.8076 (4)	1.0603 (3)	0.0403 (12)	
H28A	0.7436	0.7826	1.1165	0.06*	
H28B	0.7109	0.7983	1.0323	0.06*	
H28C	0.746	0.8686	1.0621	0.06*	
C29	1.0448 (4)	0.7550 (4)	0.8856 (3)	0.0356 (12)	
H29A	1.0678	0.7628	0.9326	0.043*	
H29B	1.0584	0.6934	0.8817	0.043*	
C30	1.1086 (4)	0.7886 (4)	0.8070 (3)	0.0489 (15)	
H30A	1.181	0.758	0.8002	0.073*	
H30B	1.098	0.8492	0.8109	0.073*	
H30C	1.0877	0.7802	0.7597	0.073*	
C31	0.8895 (4)	0.4422 (3)	0.7933 (3)	0.0260 (9)	
C32	0.9532 (4)	0.3526 (3)	0.7960 (3)	0.0354 (11)	
C33	0.9655 (5)	0.3144 (4)	0.7159 (4)	0.0593 (18)	
H33A	1.007	0.255	0.7161	0.089*	
H33B	0.9992	0.3461	0.6694	0.089*	
H33C	0.8981	0.3175	0.7106	0.089*	
C34	0.9016 (5)	0.3055 (4)	0.8705 (5)	0.067 (2)	
H34A	0.9434	0.2463	0.8725	0.101*	
H34B	0.8342	0.3074	0.8663	0.101*	
H34C	0.894	0.3323	0.9206	0.101*	
C35	1.0566 (4)	0.3539 (4)	0.8030 (4)	0.0498 (15)	
H35A	1.1009	0.2957	0.8054	0.075*	
H35B	1.0469	0.3817	0.8532	0.075*	
H35C	1.0883	0.3852	0.7551	0.075*	
C36	0.2404 (8)	0.5082 (7)	0.8503 (7)	0.092 (3)	
C37	0.2987 (11)	0.4088 (9)	0.8427 (12)	0.205 (8)	
H37A	0.3668	0.4009	0.8045	0.307*	
H37B	0.2598	0.3814	0.8221	0.307*	
H37C	0.3053	0.3832	0.8969	0.307*	
C38	0.2927 (9)	0.5410 (9)	0.9031 (10)	0.175 (7)	
H38A	0.3628	0.5383	0.8717	0.263*	
H38B	0.2934	0.5055	0.9538	0.263*	
H38C	0.2544	0.5998	0.9167	0.263*	
C39	0.2562 (9)	0.5371 (9)	0.7575 (10)	0.163 (6)	
H39A	0.3294	0.5248	0.7304	0.244*	
H39B	0.2232	0.5981	0.7524	0.244*	
H39C	0.226	0.5064	0.7315	0.244*	
C40	0.1561 (15)	0.5159 (17)	0.915 (3)	0.36 (3)	
F1	0.2531 (8)	0.9573 (4)	0.6624 (5)	0.212 (5)	
F2	0.1538 (4)	1.1178 (3)	0.6526 (3)	0.0942 (16)	
F3	0.0733 (5)	1.0160 (5)	0.7581 (6)	0.165 (3)	
F4	0.1339 (6)	1.1252 (4)	0.8083 (4)	0.138 (3)	
F5	0.3127 (5)	1.0646 (6)	0.7098 (7)	0.230 (5)	
F6	0.2306 (6)	0.9624 (4)	0.8152 (4)	0.153 (3)	
F7	0.7219 (3)	0.4491 (2)	0.6701 (3)	0.0738 (13)	
F8	0.6806 (4)	0.3849 (4)	0.8198 (3)	0.0912 (16)	
F9	0.7393 (3)	0.2881 (2)	0.6927 (3)	0.0734 (12)	
F10	0.5506 (4)	0.3231 (3)	0.7889 (3)	0.0803 (13)	
F11	0.5893 (4)	0.3878 (4)	0.6390 (3)	0.1015 (17)	
F12	0.5300 (3)	0.4862 (3)	0.7596 (3)	0.0943 (17)	
N1	0.8484 (3)	0.5115 (2)	0.7873 (2)	0.0238 (8)	
N2	0.073 (2)	0.5245 (12)	0.9253 (14)	0.285 (12)	
P1	0.60080 (8)	0.71945 (7)	0.57038 (7)	0.0193 (2)	
P2	0.90693 (9)	0.80503 (8)	0.90742 (7)	0.0234 (2)	
P3	0.65518 (9)	1.03080 (7)	0.68263 (7)	0.0220 (2)	
P4	0.47734 (9)	0.78248 (7)	0.93582 (7)	0.0229 (2)	
P5	1.03484 (8)	0.73423 (7)	0.54307 (7)	0.0213 (2)	
Re1	0.688385 (11)	0.743153 (9)	0.666496 (9)	0.01376 (4)	
Re2	0.714718 (12)	0.876729 (9)	0.714493 (9)	0.01413 (4)	
Re3	0.822021 (12)	0.777146 (10)	0.811523 (9)	0.01500 (4)	
Re4	0.795584 (12)	0.644280 (10)	0.762819 (9)	0.01549 (4)	
Re5	0.874630 (12)	0.748784 (10)	0.653646 (9)	0.01477 (4)	
Re6	0.636216 (12)	0.770712 (9)	0.824427 (9)	0.01418 (4)	
Sb1	0.19415 (3)	1.04004 (3)	0.73351 (3)	0.04855 (10)	
Sb2	0.63357 (3)	0.38715 (2)	0.72789 (2)	0.04228 (9)	
Se1	0.97085 (3)	0.65466 (3)	0.75048 (3)	0.02030 (8)	
Se2	0.74483 (3)	0.67505 (3)	0.91214 (2)	0.01991 (8)	
Se3	0.61689 (3)	0.64309 (2)	0.77419 (2)	0.01832 (8)	
Se4	0.84312 (3)	0.62241 (3)	0.61136 (2)	0.01910 (8)	
Se5	0.76775 (3)	0.84359 (3)	0.56541 (2)	0.01809 (8)	
Se6	0.89391 (3)	0.87685 (3)	0.70301 (2)	0.01902 (8)	
Se7	0.66682 (3)	0.89756 (3)	0.86628 (2)	0.01834 (8)	
Se8	0.53968 (3)	0.86534 (2)	0.72856 (2)	0.01701 (8)	

### Atomic displacement parameters (Å^2^)

**Table:**

	*U*^11^	*U*^22^	*U*^33^	*U*^12^	*U*^13^	*U*^23^
C1	0.036 (3)	0.033 (3)	0.046 (3)	0.006 (2)	−0.008 (2)	−0.011 (2)
C2	0.029 (2)	0.034 (3)	0.026 (2)	−0.004 (2)	0.0006 (19)	−0.010 (2)
C3	0.021 (2)	0.053 (3)	0.026 (2)	−0.016 (2)	−0.0067 (19)	−0.002 (2)
C4	0.024 (3)	0.093 (5)	0.040 (3)	−0.028 (3)	−0.005 (2)	0.000 (3)
C5	0.024 (2)	0.038 (3)	0.020 (2)	−0.011 (2)	0.0005 (18)	−0.0017 (19)
C6	0.040 (3)	0.035 (3)	0.034 (3)	−0.018 (2)	0.001 (2)	0.005 (2)
C7	0.043 (3)	0.021 (2)	0.031 (2)	−0.017 (2)	−0.011 (2)	0.0021 (18)
C8	0.074 (4)	0.032 (3)	0.046 (3)	−0.027 (3)	−0.030 (3)	0.000 (2)
C9	0.067 (4)	0.029 (3)	0.054 (3)	−0.013 (3)	−0.043 (3)	0.009 (2)
C10	0.032 (3)	0.020 (2)	0.054 (3)	−0.005 (2)	−0.005 (2)	−0.001 (2)
C11	0.057 (4)	0.023 (3)	0.062 (4)	0.000 (3)	−0.012 (3)	−0.001 (3)
C12	0.060 (3)	0.021 (2)	0.032 (3)	−0.013 (2)	−0.026 (2)	0.0101 (19)
C13	0.059 (4)	0.043 (3)	0.064 (4)	−0.020 (3)	−0.033 (3)	−0.013 (3)
C14	0.021 (2)	0.034 (2)	0.030 (2)	−0.0072 (19)	−0.0161 (19)	0.0001 (19)
C15	0.022 (2)	0.058 (3)	0.039 (3)	−0.017 (2)	−0.005 (2)	−0.005 (3)
C16	0.032 (3)	0.038 (3)	0.020 (2)	−0.012 (2)	−0.0107 (19)	0.0018 (19)
C17	0.037 (3)	0.052 (3)	0.025 (2)	−0.015 (3)	−0.005 (2)	−0.006 (2)
C18	0.039 (3)	0.029 (2)	0.039 (3)	−0.016 (2)	−0.021 (2)	−0.003 (2)
C19	0.029 (3)	0.050 (3)	0.020 (2)	−0.012 (2)	0.0058 (19)	−0.010 (2)
C20	0.039 (3)	0.038 (3)	0.049 (3)	−0.005 (2)	0.000 (3)	−0.019 (3)
C21	0.022 (2)	0.045 (3)	0.028 (2)	−0.017 (2)	−0.0011 (19)	−0.001 (2)
C22	0.029 (3)	0.084 (5)	0.042 (3)	−0.029 (3)	0.002 (2)	−0.004 (3)
C23	0.033 (3)	0.051 (3)	0.026 (3)	−0.016 (2)	−0.002 (2)	0.013 (2)
C24	0.062 (4)	0.043 (3)	0.052 (4)	−0.026 (3)	−0.008 (3)	0.020 (3)
C25	0.046 (3)	0.034 (3)	0.039 (3)	−0.022 (2)	−0.020 (2)	−0.004 (2)
C26	0.074 (5)	0.060 (4)	0.066 (4)	−0.034 (4)	−0.036 (4)	−0.015 (3)
C27	0.034 (3)	0.043 (3)	0.022 (2)	−0.013 (2)	−0.013 (2)	0.001 (2)
C28	0.042 (3)	0.050 (3)	0.025 (3)	−0.009 (3)	−0.005 (2)	−0.007 (2)
C29	0.026 (2)	0.057 (3)	0.030 (3)	−0.014 (2)	−0.014 (2)	−0.004 (2)
C30	0.035 (3)	0.083 (5)	0.037 (3)	−0.030 (3)	−0.007 (2)	−0.006 (3)
C31	0.030 (2)	0.027 (2)	0.020 (2)	−0.008 (2)	−0.0069 (18)	0.0022 (18)
C32	0.042 (3)	0.021 (2)	0.036 (3)	−0.001 (2)	−0.010 (2)	0.002 (2)
C33	0.068 (4)	0.044 (3)	0.063 (4)	0.012 (3)	−0.033 (4)	−0.023 (3)
C34	0.065 (4)	0.042 (4)	0.079 (5)	−0.014 (3)	−0.014 (4)	0.033 (3)
C35	0.043 (3)	0.050 (4)	0.050 (3)	0.002 (3)	−0.023 (3)	0.003 (3)
C36	0.084 (7)	0.092 (7)	0.086 (7)	−0.032 (6)	0.007 (5)	−0.013 (5)
C37	0.135 (13)	0.130 (13)	0.31 (2)	−0.012 (10)	−0.011 (14)	−0.042 (14)
C38	0.101 (10)	0.179 (14)	0.261 (19)	−0.005 (9)	−0.094 (11)	−0.043 (13)
C39	0.082 (8)	0.180 (14)	0.239 (19)	−0.045 (9)	−0.063 (10)	0.011 (13)
C40	0.097 (13)	0.149 (18)	0.71 (7)	−0.007 (13)	0.01 (2)	0.07 (3)
F1	0.334 (12)	0.087 (5)	0.122 (6)	0.063 (6)	−0.040 (7)	−0.045 (4)
F2	0.127 (4)	0.074 (3)	0.073 (3)	−0.008 (3)	−0.049 (3)	0.021 (2)
F3	0.116 (5)	0.160 (6)	0.247 (9)	−0.091 (5)	−0.062 (6)	0.048 (6)
F4	0.219 (7)	0.084 (4)	0.103 (4)	0.011 (4)	−0.076 (5)	−0.034 (3)
F5	0.082 (5)	0.226 (10)	0.394 (15)	−0.079 (6)	−0.088 (7)	0.087 (9)
F6	0.205 (7)	0.100 (4)	0.132 (5)	0.012 (5)	−0.093 (5)	0.041 (4)
F7	0.056 (2)	0.043 (2)	0.090 (3)	−0.0109 (18)	0.019 (2)	0.011 (2)
F8	0.094 (3)	0.156 (5)	0.053 (2)	−0.073 (3)	−0.015 (2)	−0.022 (3)
F9	0.095 (3)	0.0270 (17)	0.086 (3)	−0.0060 (19)	−0.016 (2)	−0.0080 (18)
F10	0.106 (3)	0.087 (3)	0.070 (3)	−0.076 (3)	−0.013 (2)	0.012 (2)
F11	0.110 (4)	0.114 (4)	0.072 (3)	−0.010 (3)	−0.035 (3)	−0.003 (3)
F12	0.045 (2)	0.060 (3)	0.154 (5)	−0.014 (2)	0.024 (3)	−0.044 (3)
N1	0.0262 (19)	0.0220 (19)	0.0246 (19)	−0.0065 (16)	−0.0097 (15)	0.0000 (15)
N2	0.49 (4)	0.151 (15)	0.29 (2)	−0.08 (2)	−0.24 (3)	0.033 (15)
P1	0.0204 (5)	0.0222 (5)	0.0198 (5)	−0.0084 (4)	−0.0097 (4)	−0.0008 (4)
P2	0.0248 (6)	0.0311 (6)	0.0203 (5)	−0.0120 (5)	−0.0106 (4)	−0.0017 (5)
P3	0.0284 (6)	0.0154 (5)	0.0244 (6)	−0.0088 (4)	−0.0091 (5)	0.0022 (4)
P4	0.0213 (5)	0.0295 (6)	0.0175 (5)	−0.0107 (5)	−0.0018 (4)	0.0010 (4)
P5	0.0172 (5)	0.0289 (6)	0.0179 (5)	−0.0080 (4)	−0.0025 (4)	−0.0028 (4)
Re1	0.01486 (7)	0.01414 (7)	0.01429 (7)	−0.00571 (6)	−0.00525 (6)	−0.00074 (6)
Re2	0.01648 (8)	0.01339 (7)	0.01429 (7)	−0.00661 (6)	−0.00464 (6)	0.00011 (6)
Re3	0.01664 (8)	0.01715 (8)	0.01394 (7)	−0.00719 (6)	−0.00573 (6)	−0.00066 (6)
Re4	0.01729 (8)	0.01348 (7)	0.01706 (8)	−0.00484 (6)	−0.00662 (6)	0.00015 (6)
Re5	0.01446 (7)	0.01705 (8)	0.01413 (7)	−0.00593 (6)	−0.00397 (6)	−0.00157 (6)
Re6	0.01552 (8)	0.01466 (7)	0.01357 (7)	−0.00644 (6)	−0.00383 (6)	0.00016 (6)
Sb1	0.0484 (2)	0.0368 (2)	0.0549 (2)	−0.00317 (17)	−0.01924 (19)	0.00604 (18)
Sb2	0.0558 (2)	0.03129 (18)	0.03719 (19)	−0.01950 (17)	−0.00054 (17)	−0.00348 (15)
Se1	0.01711 (19)	0.0226 (2)	0.0218 (2)	−0.00381 (16)	−0.00813 (16)	−0.00154 (16)
Se2	0.0243 (2)	0.0214 (2)	0.01611 (19)	−0.00918 (17)	−0.00779 (16)	0.00362 (15)
Se3	0.0207 (2)	0.01633 (19)	0.0212 (2)	−0.00994 (16)	−0.00657 (16)	0.00130 (15)
Se4	0.0200 (2)	0.01796 (19)	0.0203 (2)	−0.00409 (16)	−0.00628 (16)	−0.00549 (15)
Se5	0.0204 (2)	0.02048 (19)	0.01486 (18)	−0.00841 (16)	−0.00521 (15)	0.00141 (15)
Se6	0.0206 (2)	0.0219 (2)	0.01907 (19)	−0.01239 (16)	−0.00522 (16)	−0.00080 (16)
Se7	0.0218 (2)	0.01805 (19)	0.01662 (19)	−0.00722 (16)	−0.00446 (16)	−0.00362 (15)
Se8	0.01577 (19)	0.01644 (18)	0.01924 (19)	−0.00473 (15)	−0.00557 (15)	−0.00014 (15)

### Geometric parameters (Å, º)

**Table:**

C1—C2	1.527 (7)	C27—H27B	0.99
C1—H1A	0.98	C28—H28A	0.98
C1—H1B	0.98	C28—H28B	0.98
C1—H1C	0.98	C28—H28C	0.98
C2—P5	1.825 (5)	C29—C30	1.531 (7)
C2—H2A	0.99	C29—P2	1.830 (5)
C2—H2B	0.99	C29—H29A	0.99
C3—C4	1.529 (6)	C29—H29B	0.99
C3—P5	1.826 (5)	C30—H30A	0.98
C3—H3A	0.99	C30—H30B	0.98
C3—H3B	0.99	C30—H30C	0.98
C4—H4A	0.98	C31—N1	1.123 (6)
C4—H4B	0.98	C31—C32	1.486 (6)
C4—H4C	0.98	C32—C34	1.522 (8)
C5—C6	1.525 (7)	C32—C33	1.527 (8)
C5—P5	1.825 (5)	C32—C35	1.529 (8)
C5—H5A	0.99	C33—H33A	0.98
C5—H5B	0.99	C33—H33B	0.98
C6—H6A	0.98	C33—H33C	0.98
C6—H6B	0.98	C34—H34A	0.98
C6—H6C	0.98	C34—H34B	0.98
C7—C8	1.527 (7)	C34—H34C	0.98
C7—P3	1.831 (5)	C35—H35A	0.98
C7—H7A	0.99	C35—H35B	0.98
C7—H7B	0.99	C35—H35C	0.98
C8—H8A	0.98	C36—C40	1.36 (3)
C8—H8B	0.98	C36—C39	1.560 (16)
C8—H8C	0.98	C36—C38	1.569 (15)
C9—C12	1.525 (7)	C36—C37	1.606 (16)
C9—H9A	0.98	C37—H37A	0.98
C9—H9B	0.98	C37—H37B	0.98
C9—H9C	0.98	C37—H37C	0.98
C10—C11	1.525 (7)	C38—H38A	0.98
C10—P3	1.815 (5)	C38—H38B	0.98
C10—H10A	0.99	C38—H38C	0.98
C10—H10B	0.99	C39—H39A	0.98
C11—H11A	0.98	C39—H39B	0.98
C11—H11B	0.98	C39—H39C	0.98
C11—H11C	0.98	C40—N2	1.12 (3)
C12—P3	1.821 (5)	F1—Sb1	1.788 (6)
C12—H12A	0.99	F2—Sb1	1.851 (4)
C12—H12B	0.99	F3—Sb1	1.816 (6)
C13—C18	1.530 (6)	F4—Sb1	1.858 (5)
C13—H13A	0.98	F5—Sb1	1.791 (6)
C13—H13B	0.98	F6—Sb1	1.843 (5)
C13—H13C	0.98	F7—Sb2	1.857 (4)
C14—C15	1.528 (7)	F8—Sb2	1.871 (4)
C14—P1	1.828 (4)	F9—Sb2	1.879 (4)
C14—H14A	0.99	F10—Sb2	1.844 (4)
C14—H14B	0.99	F11—Sb2	1.804 (5)
C15—H15A	0.98	F12—Sb2	1.856 (4)
C15—H15B	0.98	N1—Re4	2.125 (4)
C15—H15C	0.98	P1—Re1	2.4741 (10)
C16—C17	1.530 (7)	P2—Re3	2.4715 (11)
C16—P1	1.827 (5)	P3—Re2	2.4729 (11)
C16—H16A	0.99	P4—Re6	2.4705 (11)
C16—H16B	0.99	P5—Re5	2.4717 (11)
C17—H17A	0.98	Re1—Se4	2.5136 (4)
C17—H17B	0.98	Re1—Se5	2.5139 (4)
C17—H17C	0.98	Re1—Se8	2.5148 (4)
C18—P1	1.824 (5)	Re1—Se3	2.5163 (4)
C18—H18A	0.99	Re1—Re4	2.6311 (2)
C18—H18B	0.99	Re1—Re6	2.6376 (3)
C19—C20	1.520 (8)	Re1—Re2	2.6396 (2)
C19—P4	1.836 (5)	Re1—Re5	2.6441 (3)
C19—H19A	0.99	Re2—Se8	2.5143 (5)
C19—H19B	0.99	Re2—Se5	2.5149 (4)
C20—H20A	0.98	Re2—Se7	2.5178 (4)
C20—H20B	0.98	Re2—Se6	2.5185 (5)
C20—H20C	0.98	Re2—Re5	2.6436 (2)
C21—C22	1.535 (6)	Re2—Re3	2.6448 (2)
C21—P4	1.830 (5)	Re2—Re6	2.6464 (2)
C21—H21A	0.99	Re3—Se7	2.5125 (4)
C21—H21B	0.99	Re3—Se1	2.5137 (4)
C22—H22A	0.98	Re3—Se2	2.5159 (4)
C22—H22B	0.98	Re3—Se6	2.5218 (4)
C22—H22C	0.98	Re3—Re4	2.6350 (3)
C23—C24	1.519 (8)	Re3—Re5	2.6393 (3)
C23—P4	1.821 (5)	Re3—Re6	2.6422 (3)
C23—H23A	0.99	Re4—Se2	2.5109 (4)
C23—H23B	0.99	Re4—Se4	2.5161 (4)
C24—H24A	0.98	Re4—Se3	2.5174 (5)
C24—H24B	0.98	Re4—Se1	2.5176 (5)
C24—H24C	0.98	Re4—Re5	2.6256 (2)
C25—C26	1.535 (7)	Re4—Re6	2.6283 (2)
C25—P2	1.825 (5)	Re5—Se5	2.5075 (4)
C25—H25A	0.99	Re5—Se6	2.5161 (4)
C25—H25B	0.99	Re5—Se4	2.5213 (4)
C26—H26A	0.98	Re5—Se1	2.5215 (4)
C26—H26B	0.98	Re6—Se8	2.5149 (4)
C26—H26C	0.98	Re6—Se3	2.5168 (4)
C27—C28	1.507 (7)	Re6—Se7	2.5186 (4)
C27—P2	1.834 (5)	Re6—Se2	2.5211 (4)
C27—H27A	0.99		
			
C2—C1—H1A	109.5	Se5—Re1—Se8	90.371 (14)
C2—C1—H1B	109.5	P1—Re1—Se3	91.74 (3)
H1A—C1—H1B	109.5	Se4—Re1—Se3	89.745 (15)
C2—C1—H1C	109.5	Se5—Re1—Se3	175.827 (14)
H1A—C1—H1C	109.5	Se8—Re1—Se3	90.025 (14)
H1B—C1—H1C	109.5	P1—Re1—Re4	134.73 (3)
C1—C2—P5	115.8 (3)	Se4—Re1—Re4	58.504 (11)
C1—C2—H2A	108.3	Se5—Re1—Re4	117.796 (12)
P5—C2—H2A	108.3	Se8—Re1—Re4	118.201 (12)
C1—C2—H2B	108.3	Se3—Re1—Re4	58.506 (11)
P5—C2—H2B	108.3	P1—Re1—Re6	135.18 (3)
H2A—C2—H2B	107.4	Se4—Re1—Re6	118.346 (11)
C4—C3—P5	116.1 (4)	Se5—Re1—Re6	118.525 (11)
C4—C3—H3A	108.3	Se8—Re1—Re6	58.374 (10)
P5—C3—H3A	108.3	Se3—Re1—Re6	58.405 (11)
C4—C3—H3B	108.3	Re4—Re1—Re6	59.847 (7)
P5—C3—H3B	108.3	P1—Re1—Re2	135.38 (3)
H3A—C3—H3B	107.4	Se4—Re1—Re2	118.501 (12)
C3—C4—H4A	109.5	Se5—Re1—Re2	58.358 (10)
C3—C4—H4B	109.5	Se8—Re1—Re2	58.332 (11)
H4A—C4—H4B	109.5	Se3—Re1—Re2	118.588 (12)
C3—C4—H4C	109.5	Re4—Re1—Re2	89.889 (8)
H4A—C4—H4C	109.5	Re6—Re1—Re2	60.196 (6)
H4B—C4—H4C	109.5	P1—Re1—Re5	134.89 (3)
C6—C5—P5	115.5 (3)	Se4—Re1—Re5	58.464 (11)
C6—C5—H5A	108.4	Se5—Re1—Re5	58.108 (10)
P5—C5—H5A	108.4	Se8—Re1—Re5	118.355 (11)
C6—C5—H5B	108.4	Se3—Re1—Re5	118.198 (11)
P5—C5—H5B	108.4	Re4—Re1—Re5	59.699 (6)
H5A—C5—H5B	107.5	Re6—Re1—Re5	89.926 (7)
C5—C6—H6A	109.5	Re2—Re1—Re5	60.045 (6)
C5—C6—H6B	109.5	P3—Re2—Se8	90.14 (3)
H6A—C6—H6B	109.5	P3—Re2—Se5	92.31 (3)
C5—C6—H6C	109.5	Se8—Re2—Se5	90.360 (14)
H6A—C6—H6C	109.5	P3—Re2—Se7	92.21 (3)
H6B—C6—H6C	109.5	Se8—Re2—Se7	89.763 (14)
C8—C7—P3	115.7 (3)	Se5—Re2—Se7	175.479 (14)
C8—C7—H7A	108.4	P3—Re2—Se6	93.99 (3)
P3—C7—H7A	108.4	Se8—Re2—Se6	175.871 (14)
C8—C7—H7B	108.4	Se5—Re2—Se6	89.567 (14)
P3—C7—H7B	108.4	Se7—Re2—Se6	89.985 (14)
H7A—C7—H7B	107.4	P3—Re2—Re1	133.58 (3)
C7—C8—H8A	109.5	Se8—Re2—Re1	58.351 (10)
C7—C8—H8B	109.5	Se5—Re2—Re1	58.321 (10)
H8A—C8—H8B	109.5	Se7—Re2—Re1	118.168 (11)
C7—C8—H8C	109.5	Se6—Re2—Re1	118.335 (11)
H8A—C8—H8C	109.5	P3—Re2—Re5	136.47 (3)
H8B—C8—H8C	109.5	Se8—Re2—Re5	118.392 (11)
C12—C9—H9A	109.5	Se5—Re2—Re5	58.103 (10)
C12—C9—H9B	109.5	Se7—Re2—Re5	118.043 (11)
H9A—C9—H9B	109.5	Se6—Re2—Re5	58.282 (11)
C12—C9—H9C	109.5	Re1—Re2—Re5	60.063 (7)
H9A—C9—H9C	109.5	P3—Re2—Re3	136.37 (3)
H9B—C9—H9C	109.5	Se8—Re2—Re3	118.169 (11)
C11—C10—P3	116.3 (4)	Se5—Re2—Re3	117.968 (12)
C11—C10—H10A	108.2	Se7—Re2—Re3	58.183 (11)
P3—C10—H10A	108.2	Se6—Re2—Re3	58.410 (11)
C11—C10—H10B	108.2	Re1—Re2—Re3	90.011 (8)
P3—C10—H10B	108.2	Re5—Re2—Re3	59.876 (7)
H10A—C10—H10B	107.4	P3—Re2—Re6	133.76 (3)
C10—C11—H11A	109.5	Se8—Re2—Re6	58.262 (10)
C10—C11—H11B	109.5	Se5—Re2—Re6	118.157 (11)
H11A—C11—H11B	109.5	Se7—Re2—Re6	58.316 (10)
C10—C11—H11C	109.5	Se6—Re2—Re6	118.306 (12)
H11A—C11—H11C	109.5	Re1—Re2—Re6	59.865 (7)
H11B—C11—H11C	109.5	Re5—Re2—Re6	89.746 (8)
C9—C12—P3	115.3 (4)	Re3—Re2—Re6	59.916 (7)
C9—C12—H12A	108.4	P2—Re3—Se7	90.34 (3)
P3—C12—H12A	108.4	P2—Re3—Se1	93.59 (3)
C9—C12—H12B	108.4	Se7—Re3—Se1	176.066 (14)
P3—C12—H12B	108.4	P2—Re3—Se2	93.07 (3)
H12A—C12—H12B	107.5	Se7—Re3—Se2	90.253 (15)
C18—C13—H13A	109.5	Se1—Re3—Se2	89.364 (15)
C18—C13—H13B	109.5	P2—Re3—Se6	91.17 (3)
H13A—C13—H13B	109.5	Se7—Re3—Se6	90.030 (15)
C18—C13—H13C	109.5	Se1—Re3—Se6	90.064 (15)
H13A—C13—H13C	109.5	Se2—Re3—Se6	175.753 (14)
H13B—C13—H13C	109.5	P2—Re3—Re4	137.20 (3)
C15—C14—P1	115.2 (3)	Se7—Re3—Re4	118.152 (12)
C15—C14—H14A	108.5	Se1—Re3—Re4	58.490 (11)
P1—C14—H14A	108.5	Se2—Re3—Re4	58.294 (11)
C15—C14—H14B	108.5	Se6—Re3—Re4	117.994 (12)
P1—C14—H14B	108.5	P2—Re3—Re5	135.51 (3)
H14A—C14—H14B	107.5	Se7—Re3—Re5	118.400 (11)
C14—C15—H15A	109.5	Se1—Re3—Re5	58.531 (10)
C14—C15—H15B	109.5	Se2—Re3—Re5	118.002 (12)
H15A—C15—H15B	109.5	Se6—Re3—Re5	58.303 (11)
C14—C15—H15C	109.5	Re4—Re3—Re5	59.712 (6)
H15A—C15—H15C	109.5	P2—Re3—Re6	134.50 (3)
H15B—C15—H15C	109.5	Se7—Re3—Re6	58.431 (11)
C17—C16—P1	115.9 (3)	Se1—Re3—Re6	118.228 (12)
C17—C16—H16A	108.3	Se2—Re3—Re6	58.456 (11)
P1—C16—H16A	108.3	Se6—Re3—Re6	118.343 (12)
C17—C16—H16B	108.3	Re4—Re3—Re6	59.741 (6)
P1—C16—H16B	108.3	Re5—Re3—Re6	89.931 (7)
H16A—C16—H16B	107.4	P2—Re3—Re2	133.10 (3)
C16—C17—H17A	109.5	Se7—Re3—Re2	58.376 (11)
C16—C17—H17B	109.5	Se1—Re3—Re2	118.558 (12)
H17A—C17—H17B	109.5	Se2—Re3—Re2	118.508 (12)
C16—C17—H17C	109.5	Se6—Re3—Re2	58.290 (11)
H17A—C17—H17C	109.5	Re4—Re3—Re2	89.692 (8)
H17B—C17—H17C	109.5	Re5—Re3—Re2	60.040 (7)
C13—C18—P1	116.6 (4)	Re6—Re3—Re2	60.072 (6)
C13—C18—H18A	108.1	N1—Re4—Se2	92.72 (10)
P1—C18—H18A	108.1	N1—Re4—Se4	90.55 (10)
C13—C18—H18B	108.1	Se2—Re4—Se4	176.729 (15)
P1—C18—H18B	108.1	N1—Re4—Se3	93.89 (10)
H18A—C18—H18B	107.3	Se2—Re4—Se3	90.179 (14)
C20—C19—P4	114.1 (3)	Se4—Re4—Se3	89.663 (14)
C20—C19—H19A	108.7	N1—Re4—Se1	89.41 (10)
P4—C19—H19A	108.7	Se2—Re4—Se1	89.388 (14)
C20—C19—H19B	108.7	Se4—Re4—Se1	90.582 (14)
P4—C19—H19B	108.7	Se3—Re4—Se1	176.691 (14)
H19A—C19—H19B	107.6	N1—Re4—Re5	132.32 (10)
C19—C20—H20A	109.5	Se2—Re4—Re5	118.699 (12)
C19—C20—H20B	109.5	Se4—Re4—Re5	58.682 (11)
H20A—C20—H20B	109.5	Se3—Re4—Re5	118.853 (11)
C19—C20—H20C	109.5	Se1—Re4—Re5	58.669 (11)
H20A—C20—H20C	109.5	N1—Re4—Re6	137.14 (10)
H20B—C20—H20C	109.5	Se2—Re4—Re6	58.701 (11)
C22—C21—P4	115.6 (4)	Se4—Re4—Re6	118.604 (11)
C22—C21—H21A	108.4	Se3—Re4—Re6	58.516 (11)
P4—C21—H21A	108.4	Se1—Re4—Re6	118.608 (12)
C22—C21—H21B	108.4	Re5—Re4—Re6	90.533 (8)
P4—C21—H21B	108.4	N1—Re4—Re1	135.52 (10)
H21A—C21—H21B	107.4	Se2—Re4—Re1	118.887 (12)
C21—C22—H22A	109.5	Se4—Re4—Re1	58.411 (11)
C21—C22—H22B	109.5	Se3—Re4—Re1	58.465 (11)
H22A—C22—H22B	109.5	Se1—Re4—Re1	119.047 (12)
C21—C22—H22C	109.5	Re5—Re4—Re1	60.396 (7)
H22A—C22—H22C	109.5	Re6—Re4—Re1	60.198 (7)
H22B—C22—H22C	109.5	N1—Re4—Re3	133.98 (10)
C24—C23—P4	116.5 (4)	Se2—Re4—Re3	58.478 (10)
C24—C23—H23A	108.2	Se4—Re4—Re3	118.890 (11)
P4—C23—H23A	108.2	Se3—Re4—Re3	118.772 (11)
C24—C23—H23B	108.2	Se1—Re4—Re3	58.346 (10)
P4—C23—H23B	108.2	Re5—Re4—Re3	60.225 (7)
H23A—C23—H23B	107.3	Re6—Re4—Re3	60.266 (7)
C23—C24—H24A	109.5	Re1—Re4—Re3	90.408 (8)
C23—C24—H24B	109.5	P5—Re5—Se5	93.02 (3)
H24A—C24—H24B	109.5	P5—Re5—Se6	90.98 (3)
C23—C24—H24C	109.5	Se5—Re5—Se6	89.788 (15)
H24A—C24—H24C	109.5	P5—Re5—Se4	93.03 (3)
H24B—C24—H24C	109.5	Se5—Re5—Se4	89.543 (14)
C26—C25—P2	116.2 (4)	Se6—Re5—Se4	175.969 (14)
C26—C25—H25A	108.2	P5—Re5—Se1	90.99 (3)
P2—C25—H25A	108.2	Se5—Re5—Se1	175.990 (15)
C26—C25—H25B	108.2	Se6—Re5—Se1	90.015 (15)
P2—C25—H25B	108.2	Se4—Re5—Se1	90.374 (15)
H25A—C25—H25B	107.4	P5—Re5—Re4	135.00 (3)
C25—C26—H26A	109.5	Se5—Re5—Re4	118.239 (12)
C25—C26—H26B	109.5	Se6—Re5—Re4	118.553 (12)
H26A—C26—H26B	109.5	Se4—Re5—Re4	58.488 (11)
C25—C26—H26C	109.5	Se1—Re5—Re4	58.525 (11)
H26A—C26—H26C	109.5	P5—Re5—Re3	133.52 (3)
H26B—C26—H26C	109.5	Se5—Re5—Re3	118.448 (12)
C28—C27—P2	116.3 (4)	Se6—Re5—Re3	58.511 (10)
C28—C27—H27A	108.2	Se4—Re5—Re3	118.534 (11)
P2—C27—H27A	108.2	Se1—Re5—Re3	58.245 (11)
C28—C27—H27B	108.2	Re4—Re5—Re3	60.063 (6)
P2—C27—H27B	108.2	P5—Re5—Re2	135.05 (3)
H27A—C27—H27B	107.4	Se5—Re5—Re2	58.377 (11)
C27—C28—H28A	109.5	Se6—Re5—Re2	58.369 (11)
C27—C28—H28B	109.5	Se4—Re5—Re2	118.063 (12)
H28A—C28—H28B	109.5	Se1—Re5—Re2	118.314 (12)
C27—C28—H28C	109.5	Re4—Re5—Re2	89.919 (8)
H28A—C28—H28C	109.5	Re3—Re5—Re2	60.083 (6)
H28B—C28—H28C	109.5	P5—Re5—Re1	136.44 (3)
C30—C29—P2	115.2 (4)	Se5—Re5—Re1	58.345 (11)
C30—C29—H29A	108.5	Se6—Re5—Re1	118.252 (11)
P2—C29—H29A	108.5	Se4—Re5—Re1	58.179 (10)
C30—C29—H29B	108.5	Se1—Re5—Re1	118.413 (12)
P2—C29—H29B	108.5	Re4—Re5—Re1	59.905 (6)
H29A—C29—H29B	107.5	Re3—Re5—Re1	90.032 (7)
C29—C30—H30A	109.5	Re2—Re5—Re1	59.892 (7)
C29—C30—H30B	109.5	P4—Re6—Se8	91.62 (3)
H30A—C30—H30B	109.5	P4—Re6—Se3	90.53 (3)
C29—C30—H30C	109.5	Se8—Re6—Se3	90.011 (14)
H30A—C30—H30C	109.5	P4—Re6—Se7	93.56 (3)
H30B—C30—H30C	109.5	Se8—Re6—Se7	89.731 (14)
N1—C31—C32	174.3 (5)	Se3—Re6—Se7	175.909 (14)
C31—C32—C34	109.2 (4)	P4—Re6—Se2	92.58 (3)
C31—C32—C33	107.7 (4)	Se8—Re6—Se2	175.794 (14)
C34—C32—C33	111.8 (5)	Se3—Re6—Se2	89.961 (15)
C31—C32—C35	107.0 (4)	Se7—Re6—Se2	89.997 (15)
C34—C32—C35	110.5 (5)	P4—Re6—Re4	134.44 (3)
C33—C32—C35	110.6 (5)	Se8—Re6—Re4	118.303 (12)
C32—C33—H33A	109.5	Se3—Re6—Re4	58.538 (11)
C32—C33—H33B	109.5	Se7—Re6—Re4	118.180 (12)
H33A—C33—H33B	109.5	Se2—Re6—Re4	58.323 (11)
C32—C33—H33C	109.5	P4—Re6—Re1	133.65 (3)
H33A—C33—H33C	109.5	Se8—Re6—Re1	58.370 (10)
H33B—C33—H33C	109.5	Se3—Re6—Re1	58.386 (10)
C32—C34—H34A	109.5	Se7—Re6—Re1	118.212 (11)
C32—C34—H34B	109.5	Se2—Re6—Re1	118.266 (12)
H34A—C34—H34B	109.5	Re4—Re6—Re1	59.954 (6)
C32—C34—H34C	109.5	P4—Re6—Re3	136.23 (3)
H34A—C34—H34C	109.5	Se8—Re6—Re3	118.242 (11)
H34B—C34—H34C	109.5	Se3—Re6—Re3	118.521 (11)
C32—C35—H35A	109.5	Se7—Re6—Re3	58.208 (10)
C32—C35—H35B	109.5	Se2—Re6—Re3	58.265 (11)
H35A—C35—H35B	109.5	Re4—Re6—Re3	59.993 (7)
C32—C35—H35C	109.5	Re1—Re6—Re3	90.109 (7)
H35A—C35—H35C	109.5	P4—Re6—Re2	135.72 (3)
H35B—C35—H35C	109.5	Se8—Re6—Re2	58.239 (11)
C40—C36—C39	132.9 (19)	Se3—Re6—Re2	118.312 (12)
C40—C36—C38	90.2 (19)	Se7—Re6—Re2	58.286 (11)
C39—C36—C38	119.0 (10)	Se2—Re6—Re2	118.257 (12)
C40—C36—C37	106.9 (15)	Re4—Re6—Re2	89.802 (8)
C39—C36—C37	100.2 (10)	Re1—Re6—Re2	59.939 (6)
C38—C36—C37	105.2 (11)	Re3—Re6—Re2	60.012 (6)
C36—C37—H37A	109.5	F1—Sb1—F5	89.4 (5)
C36—C37—H37B	109.5	F1—Sb1—F3	91.1 (5)
H37A—C37—H37B	109.5	F5—Sb1—F3	179.5 (5)
C36—C37—H37C	109.5	F1—Sb1—F6	88.7 (3)
H37A—C37—H37C	109.5	F5—Sb1—F6	90.7 (4)
H37B—C37—H37C	109.5	F3—Sb1—F6	89.3 (4)
C36—C38—H38A	109.5	F1—Sb1—F2	91.6 (3)
C36—C38—H38B	109.5	F5—Sb1—F2	90.8 (3)
H38A—C38—H38B	109.5	F3—Sb1—F2	89.1 (3)
C36—C38—H38C	109.5	F6—Sb1—F2	178.4 (3)
H38A—C38—H38C	109.5	F1—Sb1—F4	179.2 (3)
H38B—C38—H38C	109.5	F5—Sb1—F4	90.3 (5)
C36—C39—H39A	109.5	F3—Sb1—F4	89.1 (4)
C36—C39—H39B	109.5	F6—Sb1—F4	90.6 (3)
H39A—C39—H39B	109.5	F2—Sb1—F4	89.1 (2)
C36—C39—H39C	109.5	F11—Sb2—F10	91.2 (2)
H39A—C39—H39C	109.5	F11—Sb2—F12	89.6 (2)
H39B—C39—H39C	109.5	F10—Sb2—F12	90.7 (2)
N2—C40—C36	138 (5)	F11—Sb2—F7	91.7 (2)
C31—N1—Re4	170.0 (4)	F10—Sb2—F7	176.8 (2)
C18—P1—C16	105.1 (2)	F12—Sb2—F7	90.78 (17)
C18—P1—C14	104.4 (2)	F11—Sb2—F8	179.2 (3)
C16—P1—C14	101.1 (2)	F10—Sb2—F8	88.6 (2)
C18—P1—Re1	113.18 (16)	F12—Sb2—F8	91.2 (2)
C16—P1—Re1	115.75 (16)	F7—Sb2—F8	88.5 (2)
C14—P1—Re1	115.83 (15)	F11—Sb2—F9	89.3 (2)
C25—P2—C29	105.2 (3)	F10—Sb2—F9	90.5 (2)
C25—P2—C27	105.3 (2)	F12—Sb2—F9	178.4 (2)
C29—P2—C27	100.2 (2)	F7—Sb2—F9	88.10 (17)
C25—P2—Re3	112.62 (16)	F8—Sb2—F9	89.9 (2)
C29—P2—Re3	116.10 (16)	Re3—Se1—Re4	63.164 (12)
C27—P2—Re3	115.91 (16)	Re3—Se1—Re5	63.224 (11)
C10—P3—C12	105.2 (3)	Re4—Se1—Re5	62.806 (11)
C10—P3—C7	105.7 (2)	Re4—Se2—Re3	63.228 (11)
C12—P3—C7	101.1 (2)	Re4—Se2—Re6	62.976 (11)
C10—P3—Re2	113.02 (16)	Re3—Se2—Re6	63.279 (11)
C12—P3—Re2	114.80 (16)	Re1—Se3—Re6	63.209 (11)
C7—P3—Re2	115.75 (16)	Re1—Se3—Re4	63.028 (11)
C23—P4—C21	105.2 (2)	Re6—Se3—Re4	62.945 (10)
C23—P4—C19	101.2 (2)	Re1—Se4—Re4	63.084 (10)
C21—P4—C19	104.5 (2)	Re1—Se4—Re5	63.358 (11)
C23—P4—Re6	116.11 (18)	Re4—Se4—Re5	62.830 (10)
C21—P4—Re6	113.55 (15)	Re5—Se5—Re1	63.547 (12)
C19—P4—Re6	114.80 (16)	Re5—Se5—Re2	63.520 (11)
C2—P5—C5	101.4 (2)	Re1—Se5—Re2	63.320 (11)
C2—P5—C3	105.3 (2)	Re5—Se6—Re2	63.349 (10)
C5—P5—C3	105.4 (2)	Re5—Se6—Re3	63.186 (11)
C2—P5—Re5	114.95 (17)	Re2—Se6—Re3	63.301 (11)
C5—P5—Re5	115.96 (15)	Re3—Se7—Re2	63.440 (10)
C3—P5—Re5	112.57 (15)	Re3—Se7—Re6	63.361 (11)
P1—Re1—Se4	91.41 (3)	Re2—Se7—Re6	63.399 (10)
P1—Re1—Se5	92.39 (3)	Re2—Se8—Re1	63.317 (11)
Se4—Re1—Se5	89.573 (15)	Re2—Se8—Re6	63.499 (11)
P1—Re1—Se8	92.54 (3)	Re1—Se8—Re6	63.256 (11)
Se4—Re1—Se8	176.044 (14)		
